# A metagenomic approach to unveil the association between fecal gut microbiota and short-chain fatty acids in diarrhea caused by diarrheagenic *Escherichia coli* in children

**DOI:** 10.15698/mic2024.04.820

**Published:** 2024-04-30

**Authors:** Pablo Gallardo, Mariana Izquierdo, Tomeu Viver, Esteban Bustos-Caparros, Dana Piras, Roberto M. Vidal, Hermie J.M. Harmsen, Mauricio J. Farfan

**Affiliations:** 1 Department of Medical Microbiology and Infection prevention, University of Groningen, University Medical Center Groningen, Groningen, The Netherlands.; 2 Departamento de Cirugía y Pediatría Oriente, CICA Hospital Dr. Luis Calvo Mackenna, Facultad de Medicina, Universidad de Chile, Santiago, Chile.; 3 Marine Microbiology Group, Department of Animal and Microbial Diversity, Mediterranean Institute of Advanced Studies (CSIC-UIB), Esporles, Illes Balears, Spain.; 4 Programa de Microbiología y Micología, Instituto de Ciencias Biomédicas, Facultad de Medicina, Universidad de Chile, Santiago, Chile.

**Keywords:** diarrheagenic Escherichia coli, short-chain fatty acids (SCFAs), fecal gut microbiota, metagenomics, diarrhea in children

## Abstract

Diarrheagenic *Escherichia coli* (DEC) is the main cause of diarrhea in children under five years old. The virulence of DEC is tightly regulated by environmental signals influenced by the gut microbiota and its metabolites. Short-chain fatty acids (SCFAs) are the main metabolic product of anaerobic fermentation in the gut, but their role in DEC diarrhea has not yet been established. In this study, we determine the levels of acetate, propionate, and butyrate in stool samples from children with diarrhea caused by DEC, and we identify bacteria from the fecal gut microbiota associated with the production of SCFAs. The microbiota and SCFAs levels in stool samples obtained from 40 children with diarrhea and 43 healthy children were determined by 16S rRNA gene sequencing and HPLC, respectively. Additionally, shotgun metagenomics was used to identify metagenome-assembled genomes (MAGs) in a subgroup of samples. The results showed significantly higher levels of all SCFAs tested in diarrheal samples than in healthy controls. The abundance of *Streptococcus* sp., *Limosilactobacillus*, *Blautia*, *Escherichia*, *Bacteroides*, *Megamonas,* and *Roseburia* was higher in the DEC group than in the healthy individuals. Functional analysis of bacteria and their main metabolic pathways made it possible to identify species MAGs that could be responsible for the detected SCFAs levels in DEC-positive diarrhea. In conclusion, based on our results and published data, we suggest that SCFAs may be important in the crosstalk between the microbiota and DEC pathogens in the gut.

## INTRODUCTION

The environment and/or bacterial regulators play a significant role in the highly regulated process of virulence gene expression in enteropathogens [1–3]. Under well-defined environmental conditions, the expression of virulence genes occurs at a specific site, allowing bacteria to initiate the infection process [4]. The resident microbiota and its metabolites strongly regulate the environment that pathogens like diarrheagenic *Escherichia coli* (DEC) encounter when they colonize the intestinal mucosa [4–6]. Several reports have demonstrated that compared to healthy children, a distinctive fecal gut microbiota is found in children suffering from diarrhea, such as those associated with infection by DEC pathotypes [7, 8]. The changes in microbiota composition have been linked to metabolites found in the intestinal tract that may favor the interactions between the intestinal microbiota and the infecting pathogen [8]. Compounds such as mucin, succinate, hydrogen, bile acids, autoinducers, and short-chain fatty acids (SCFAs) have been implicated in this crosstalk between the microbiota and the pathogen [9].

SCFAs are generated through the fermentation of dietary fibers, mainly by anaerobic bacteria in the gut microbiota [10]. The primary SCFAs in the human intestine include acetate, propionate, and butyrate. The concentrations of these molecules vary along the gastrointestinal tract, from lower concentrations in the ileum (20–40 mM) to higher concentrations in the cecum and proximal colon (70–140 mM) [11, 12]. SCFAs play key roles in many physiological processes, such as lipid metabolism, appetite regulation, and immune function. Also, SCFAs are important molecular signals between the microbiota and the host [13–15]. Several enteropathogens, such as *Campylobacter jejuni*, *Shigella* spp., *Salmonella* spp., *Listeria monocytogenes,* and DEC, can sense and respond to SCFAs [16]; however, the evidence obtained so far is controversial. Some reports suggest that SCFAs might protect the host against enteropathogens, while others have demonstrated that virulence is closely related to SCFAs levels, inducing the expression of virulence factors [17, 18].

SCFAs production involves active enzymatic pathways in some intestinal bacterial groups [19]. Metagenomic and culturomic approaches have facilitated identifying and characterizing bacteria responsible for SCFAs production. Acetate, the most abundant SCFAs in the gut, is produced by most enteric bacteria, including *Akkermansia*, *Bacteroides*, *Bifidobacterium*, *Prevotella*, *Ruminococcus*, *Blautia*, *Clostridium,* and *Streptococcus.* Unlike acetate, butyrate and propionate production is more conserved and restricted to specific genera. Butyrate-producing species belong to *Faecalibacterium*, *Eubacterium*, *Roseburia*, *Anaerostipes*, *Coprococcus* and *Subdoligranulum* genera, while propionate-producing bacteria belong to *Bacteroides*, *Prevotella*, *Alistipes*, *Roseburia*, *Eubacterium*, *Blautia*, *Coprococcus*, *Phascolarctobacterium,* and *Akkermansia* [20–22].

Despite SCFAs having been linked to regulating enteropathogen virulence, little is known about the number of SCFAs present during diarrheal episodes caused by DEC, particularly in young children. In this study, we sought to determine the SCFAs levels in stool samples from children with diarrhea by DEC and to identify the bacterial species from fecal gut microbiota associated with the production of SCFAs. Our results increase the knowledge of the association between SCFAs during diarrhea and changes in the microbiota composition associated with the presence of DEC pathogens.

## RESULTS

### SCFAs levels in stool samples from the DEC and the healthy groups

**Figure 1:**
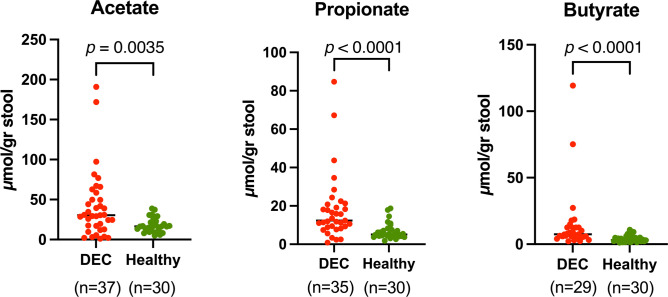
FIGURE 1: Levels of SCFAs in stool samples. Stool sample concentrations of acetate, propionate, and butyrate were identified using high-performance liquid chromatography (HPLC). Red dots represent DEC-positive samples, while green dots represent samples from healthy children. Values are normalized by dry pellet weight. Analysis was performed using the Mann-Whitney test. The p-values for each SCFAs are displayed in the figure. The number of samples (n) included in each group is stated in parentheses.

We measured the levels of acetate, propionate, and butyrate in stool samples of the DEC (40 samples) and healthy (43 samples) groups and found significant differences in all SCFAs tested **(Figure 1)**. For acetate, median values were 30.4 (15.1-54.3) µmol/gr for samples in the DEC group compared to 16.5 (11.0-23.8) µmol/g for the healthy group. For propionate and butyrate, the median (IQR) values were 12.9 (8.9-20.8) µmol/g and 7.4 (5.7-12.6) µmol/g for samples in the DEC group, compared to 5.1 (4.1-7.7) µmol/g and 3.1 (2.2-5.2) µmol/g for the healthy group, respectively. Therefore, all measured SCFAs from DEC-positive diarrheal samples were higher than those from healthy controls. When comparing the levels of SCFAs within DEC samples based on pathotypes, elevated levels of the three measured SCFAs in EPEC samples were noted compared to healthy controls. Furthermore, we found higher levels of acetate and propionate in STEC samples compared to healthy controls. For EAEC, no difference of SCFAs compared to the healthy controls was found (Supplementary Figure 1).

### SCFA-producing genera are present in the fecal gut microbiota

**Figure 2:**
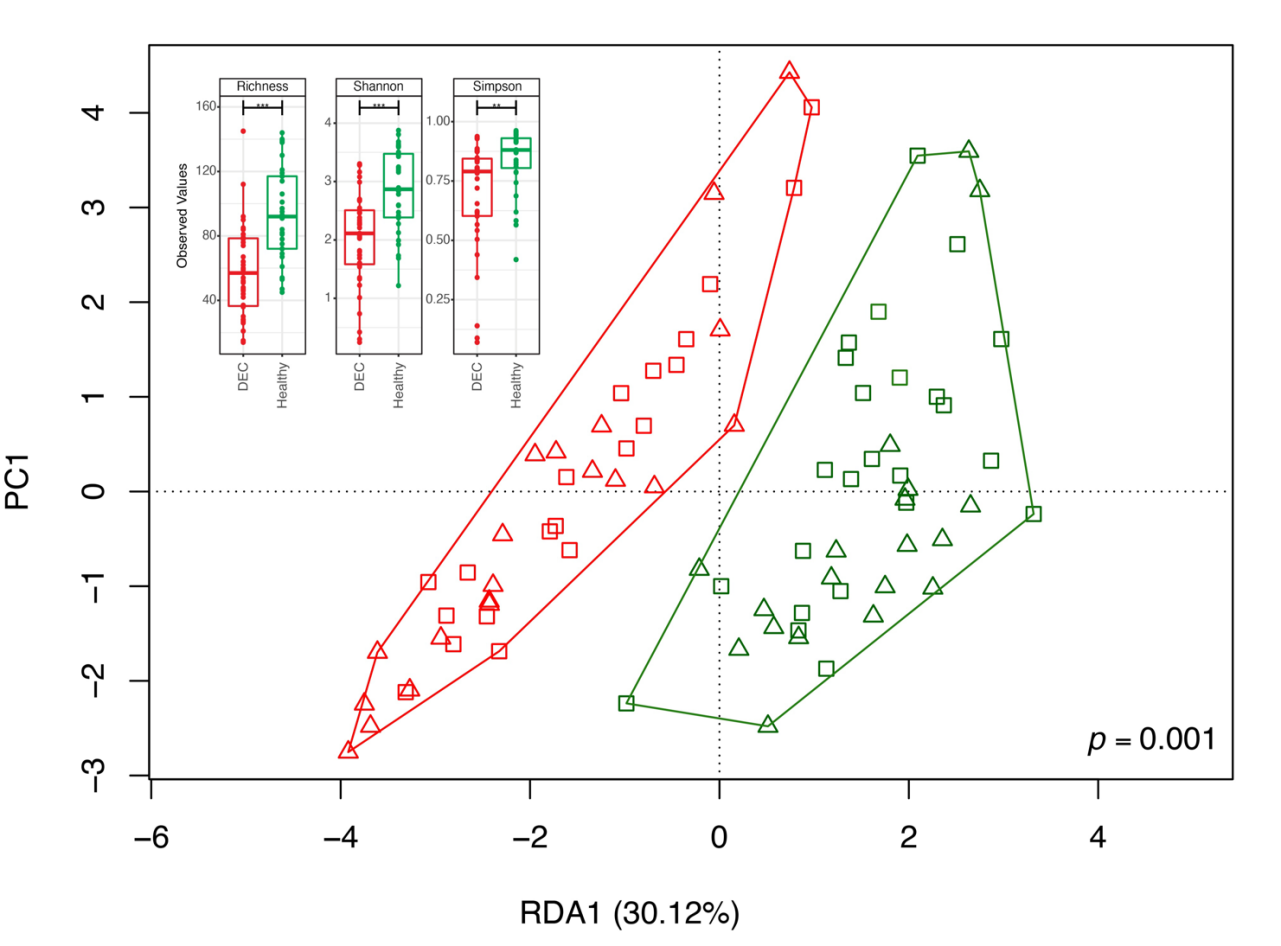
FIGURE 2: Community structure DEC-positive and healthy stool samples and diversity indexes. The distribution of the microbial community in each sample and its clustering based on the sample group were evaluated using a redundancy analysis (RDA). Red and green symbols represent the microbiota compositions observed in the DEC and healthy groups, respectively. Triangles represent samples of children under 3 years old and squares of children between 3 and 5 years of age. The analysis used a sample classification as the explanatory matrix and relative ASV abundance as the response matrix. The data were normalized with a double square root transformation. The clustering significance of the RDA was assessed by ANOVA, utilizing the *vegan* package for R. The subplot illustrates the variety of alpha diversity indexes of samples for both the DEC and healthy groups.

**Figure 3:**
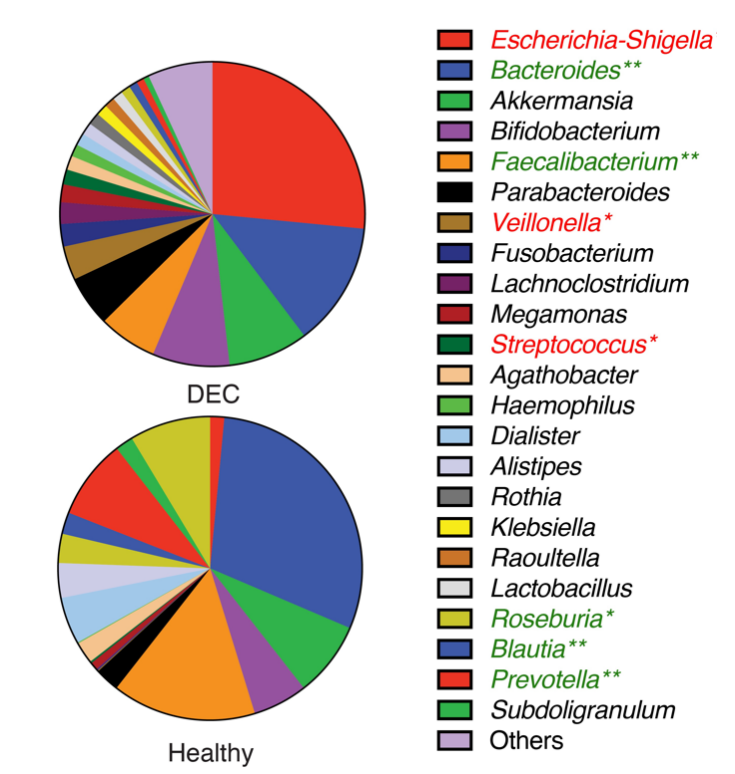
FIGURE 3: Genera abundance of DEC and healthy samples. The classification of ASVs was done using the SILVA 138 database as a reference. Relative abundance of the main genera (abundance > 1%) found in both groups. Genera with an abundance lower than 1% are grouped as “others.” Significant genera were determined using the Mann-Whitney U test. Significance is indicated (**p < 0.01, *p < 0.05).

To identify specific genera associated with the levels of SCFAs found in the stool samples, we first determined the fecal gut microbiota composition by 16S rRNA gene analysis from over 20,000 sequences per sample (Supplementary Table 1). A total of 2,537 amplicon sequence variants (ASVs) were obtained between both groups and reduced to 459 unique taxonomic classifications. Diversity analysis showed lower alpha diversity in the DEC group than in the healthy group **(Figure 2)**. The redundancy analysis (RDA) of the ASV data showed a different microbiota composition and community structure between the DEC and healthy groups, with clear group clustering. No differences in the distribution of samples within groups was observed when age levels was evaluated **(Figure 2)**. Regarding the *Firmicutes/Bacteroidota* ratio, we observed a significant difference in this ratio between the healthy (1.15 ±.0.49) and DEC group (7.2 ±1.53) (Supplementary Figure 2). Of all SCFA-producing genera, a significant abundance of the genera *Escherichia-Shigella* and *Streptococcus* was found in the DEC group compared to the healthy group using both, the Mann-Whitney non-parametric method and differential analysis; the genera *Faecalibacterium*, *Bacteroides*, *Blautia,* and *Ruminococcus* were significantly higher in the healthy group than in the DEC group only with the non-parametric approach (**Figure 3**, Supplementary Figure 3). Consequently, we identified specific genera that can produce SCFAs associated with DEC-positive diarrheal stool samples.

### Correlation between SCFA levels with fecal gut microbiota

**Figure 4:**
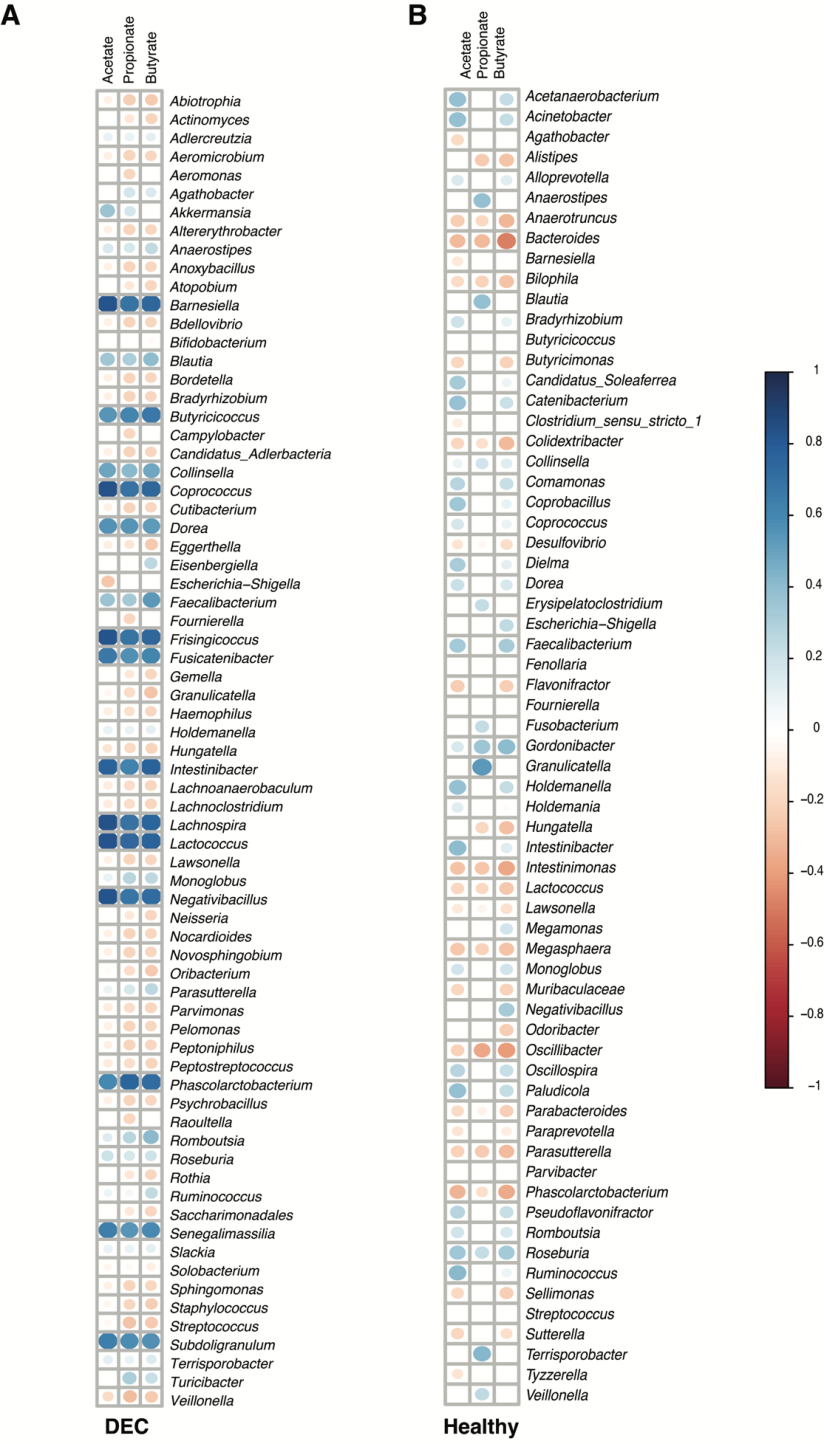
FIGURE 4: Correlation between identified genera and detected levels of SCFAs. Detected acetate, propionate, and butyrate levels within DEC (A) and Healthy (B) groups were correlated using *Pearson* with the relative abundance of the main genera in each group. Only significant (*p* < 0.05) correlations are shown; non-significant correlations are shown as empty squares. The intensity of correlation is indicated by the shifting in colors and size of the circles.

We further explored the correlations between the relative abundance of the gut microbiota and SCFA levels in stool samples from the DEC and healthy groups. The heatmap representing *Pearson* correlations for the abundance of differential bacteria found in the DEC group and quantified SCFAs in feces revealed that levels of acetate, propionate, and butyrate were positively correlated with the abundance of *Faecalibacterium*, *Roseburia*, *Blautia,* and *Subdoligranulum*; the *Akkermansia* genera abundance was only positively correlated with butyrate levels. Regarding negative associations in the DEC group, a negative correlation with the abundance of the genera *Veillonella*, *Streptococcus*, and *Haemophilus* was found with the fecal levels of acetate and propionate. In addition, the genus *Raoultella* was negatively correlated with propionate fecal levels **(Figure 4A)**. *Faecalibacterium* abundance was positively correlated with all three SCFAs in the healthy group. In addition, a positive correlation was found between *Roseburia* genus abundance and propionate and butyrate levels. *Bacteroides* genus abundance was negatively correlated with fecal levels of all tested SCFAs **(Figure 4B)**. Our data suggest a possible association between SCFA levels in stool samples and the bacterial genera identified from the fecal gut microbiota.

### Correlation between fecal SCFA levels and bacterial species identified from assembled genomes

Shotgun metagenomics was used to identify metagenome-assembled genomes (MAGs) in a subgroup of samples. Clustering based on microbiota composition (Supplementary Figure 4) and fecal SCFA levels (Supplementary Figure 5) confirmed the representability of the selected samples for metagenomic analysis. The analysis of metagenomes using Nonpareil showed a higher sequencing coverage (C) in DEC samples and higher sequence diversity (*Nd*) in healthy samples compared to DEC, results that mirrored the diversity observed by 16S sequencing (Supplementary Figure 6 A-B). The expected sequencing effort for 95% of coverage was not significantly different from what was observed (Supplementary Figure 6C). The binning of contigs from metagenomes resulted in 659 MAGs. After checking for completeness and contamination, 309 MAGs were used in the dereplication process. Of these, 63 were unique MAGs, and 54 were genospecies (Supplementary Table 2). Biological and statistical associations between MAGs and fecal levels of SCFAs using MetOrigin showed at the genera level that the higher levels of acetate in the DEC group compared to the healthy group were positively associated with the high presence of *Streptococcus*, *Bacteroides*, *Megamonas*, *Escherichia,* and *Limosilactobacillus,* as well as the decrease of *Lactobacillus*, *Bifidobacterium,* and *Ruminococcus* in DEC samples compared to the healthy group. On the other hand, higher levels of propionate in the DEC group were also related to the presence of *Streptococcus*, *Bacteroides*, *Escherichia*, *Limosilactobacillus,* and a decrease in *Ruminococcus*. Other genera involved in the production of acetate and butyrate, which were less abundant in the DEC group, were negatively associated with high SCFA levels (Supplementary Figure 7).

**Figure 5:**
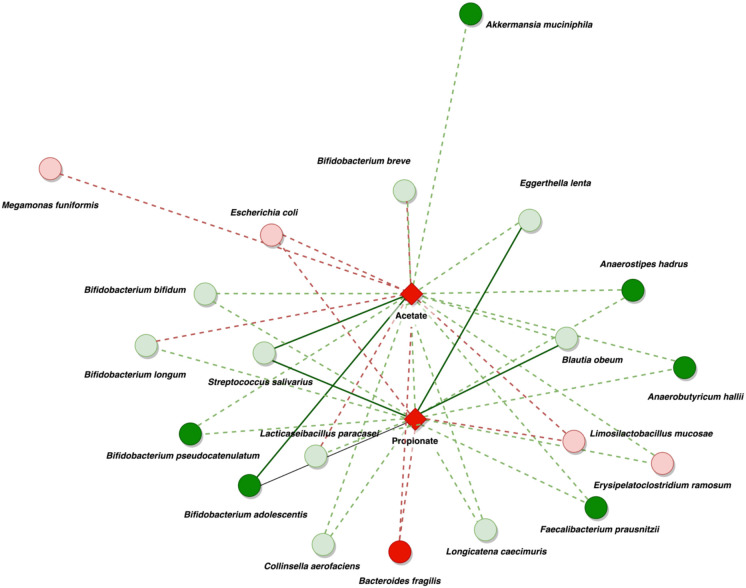
FIGURE 5: Summary of network co-metabolism in DEC samples at the species level. The network illustrates the relationships between bacteria and significant SCFAs in DEC samples. The circles in the diagram symbolize bacteria. The red circles indicate highly abundant bacteria, with a soft red color indicating non-significant abundance and bold red indicating significant abundance. The green circles represent a decrease in bacterial presence, with a soft green color indicating a non-significant decrease and bold green indicating a significant reduction when comparing DEC samples to healthy individuals. Diamonds symbolize metabolites (bold red for significant presence). The biological associations are represented by red and green lines, with red indicating positive and green indicating negative associations. Soft lines suggest non-significant associations, while strong lines imply significant associations.

Positive associations were reduced and constrained to fewer organisms at the species level. This analysis showed positive associations between acetate and propionate fecal levels in the DEC group, principally with the presence of the following species in the DEC group: *Bacteroides fragilis*, *Limosilactobacillus mucosae,* and *Escherichia coli*. Additional positive associations were observed between acetate levels and the presence of *Megamonas funiformis* and *Bifidobacterium breve*. The presence of *Bifidobacterium longum* in the DEC group compared with that in the healthy group was also negatively correlated with the levels of acetate in the DEC group. The presence of *Blautia* species, *Anaerostipes hadrus*, *Anaerobutyricum hallii*, *Faecalibacterium prausnitzii,* and some other *Bifidobacterium* species was negatively associated with the high levels of acetate and butyrate detected in the DEC group **(Figure 5)**. Although specific bacteria were associated with the detected levels of SCFAs, no correlations were found with the presence of terminal genes involved in the bacterial production of acetate, propionate, and butyrate (Supplementary Figure 8). Our data highlight specific bacterial species from the gut microbiota associated with the levels of SCFAs in DEC-positive diarrhea.

## DISCUSSION

Changes in microbiota composition have been described in several infectious diseases, including diarrhea [23]. In previous studies, we highlighted the distinctive microbiota associated with DEC-positive diarrhea in children under 5 years of age and the potential metabolic environment associated with the disease [7, 8]. To further study the metabolic environment associated with diarrhea, which might be involved in DEC pathogenicity, we focused on the role of acetate, propionate, and butyrate, the most abundant SCFAs in the gut. These metabolites, synthesized by the gut microbiota, have been linked to human health and disease [21, 23–25]. Considering that the concentration of SCFAs varies along the gastrointestinal tract, it is unsurprising that pathogens can sense these molecules to trigger the expression of virulence factors in enteric pathogens along the gastrointestinal tract. In this sense, several groups have shown that SCFAs might act as signaling molecules for several enteropathogens, regulating the expression of adhesins, mobility, and virulence regulators [18, 26]. For DEC, the role of SCFAs in DEC pathogenicity has been evaluated, and some mechanisms have been proposed [16, 18, 27–29], but the involvement of the gut microbiota of children remains unclear.

When comparing the detected levels within diarrheal samples against the control group, a significantly higher amount of all SCFAs tested was found in the DEC samples **(Figure 1)**. Regarding DEC pathotypes, higher levels of the three measured SCFAs on EPEC samples were found compared to healthy controls. Furthermore, we found higher levels of acetate and propionate in STEC samples compared to healthy controls (Supplementary Figure 1). Fecal SCFAs in adult patients suffering from diarrhea have been reported; in patients suffering from infection by *Vibrio cholerae,* there were higher levels of SCFAs than in patients with diarrhea negative for this pathogen [30]. Meanwhile, fecal SCFA levels of adult patients with bile acid diarrhea and irritable bowel syndrome (IBS) and healthy controls are not significantly different in the amounts of acetate, propionate, and butyrate [31]; adult patients with antibiotic-associated diarrhea present significantly lower amounts of SCFAs than healthy controls [32]. A study on obesity-linked gut microbiota dysbiosis to high levels of SCFAs, with higher levels in individuals with diarrheal stools and lower levels in normal or solid stools. Higher SCFA levels were positively associated with gut microbiome dysbiosis and gut permeability [14]. These findings on SCFAs and dysbiosis seem to be in line with our results.

To demonstrate dysbiosis in comparison to healthy children, our and other research groups have concentrated on identifying changes in the gut microbiota in children with diarrhea [7, 8, 33, 34]. In this work, the fecal gut microbiota composition by 16S rRNA gene analysis reveals a lower beta diversity was observed within the diarrheal group compared to the healthy samples **(Figure 2)**, in addition to a significant difference in the *Firmicutes/Bacteroidetes* ratio (Supplementary Figure 2). To identify specific species with the capability to produce SCFAs, a metagenome analysis of a subgroup of samples was performed. Recovered MAGs mostly belong to *Firmicutes*, bacteria involved in acetate production through the Wood-Ljungdahl pathway or from pyruvate via acetyl-CoA *Streptococcus, Anaerobutyricum, Ruminococcus* [35], and propionate producers such as *Akkermansia, Roseburia,* and *Bacteroides* [36]. Regarding butyrate production, we acknowledge *Faecalibacterium prausnitzii* and other producers, which also produce acetate and propionate. This multirole complicates the association of specific species with the detected SCFA levels. Our data also show that the expected contribution of specific genera to the levels of SCFAs can differ from what could be determined at the species level, possibly due to the absence of specific genes in some organisms, as observed with *Streptococcus* and *Streptococcus salivarius* from assembled genomes and their association with the production of acetate and propionate **(Figure 5)**. Previous studies have highlighted the role of *S. salivarius* strains as new potential probiotics; metabolites produced by these organisms protected mouse fibroblasts against oxidative stress, exhibited antimicrobial activity, and had an antiproliferative effect on liver and breast cancer [37] or inhibited the activation of the NF-kB pathway in HT-29 cells [38]. However, this effect could not be attributed to any specific metabolite. Furthermore, a negative correlation was observed between SCFA levels and these species. In bacteria associated with fecal levels of acetate and propionate in DEC samples, there is no unique role for these organisms. For example, *Megamonas funiformis* has been defined as a harmful bacterium due to its opportunistic presence in inflammatory conditions [39], while *Limosilactobacillus mucosae* (formerly known as *Lactobacillus mucosae*) has been classified as a potential probiotic due to its ability to adhere to the intestinal epithelium and protect against pathogens like *E. coli* and increased butyrate levels in piglet stool samples [40, 41]. In the case of *E. coli*, this bacterium excretes acetate upon growth on fermentable sugars, but it can switch the *Pta-AckA* pathway from production to consumption, co-consuming glucose and acetate under excess glucose [42]. It is important to note that the recovered MAGs identified as *E. coli* were associated with commensal species and not phylogenetically related to DEC. A recent study evaluated the effect of SCFAs on 140 pathogenic *E. coli* isolates, finding that they restored the susceptibility of almost all isolates to tested β-lactams and inhibited motility. The expression of bacterial genes under colonic conditions was reduced or suppressed concentration-dependent, but lower concentrations resulted in increased expression of all evaluated genes [28].

SCFAs play a crucial role in immune modulation during gut inflammation, as dysbiosis and inflammation can compromise barrier integrity, creating opportunities for pathogens and cellular damage. SCFAs can modulate immune responses through G protein-coupled receptors (GPR), toll-like receptors (TLR), and their associated signaling cascades [43, 44]. However, several studies have reported contradictory results, with some showing that butyrate increases the sensitivity of intestinal cell lines to Shiga toxin. In contrast, others show dose-dependent protective and non-protective responses [24, 45]. Several mechanisms could account for the high fecal SCFAs levels in diarrheal samples compared to healthy samples found in this study. We know that diarrhea is characterized by inflammation of epithelia and less absorption of metabolites, which could also lead to increased SCFA levels in stool samples [46]**.** We have observed changes in the microbiota and the diarrheal process itself that increase the levels of some SCFAs in the colon, especially acetate, which could modulate the immune response and virulence of pathogens in the gut, but a better approach might involve including other metabolites, such as lactate, valerate, and isovalerate, which are interconnected in the bacterial production of acetate, propionate, and butyrate. Another possible mechanism might be related to gene expression in producing SCFAs. A previous study using metagenomic data from non-industrialized and industrialized populations reported differences in the abundance of genes responsible for SCFAs [47]. Using our metagenomic data, we found no differences between the DEC and healthy groups in the abundance of terminal genes for producing acetate, propionate, and butyrate (Supplementary Figure 8). We acknowledge that transcriptomic data from these samples would be useful to determine the real impact of the evaluated genes and organisms present; however, this analysis was not possible considering the storage conditions of the samples in our study. Finally, it is important to note that the concentration of SCFAs produced by the gut microbiota in the colon is influenced by the absorption of these molecules and the fluid and electrolyte imbalance during diarrhea episodes [48]. The mechanism(s) that could explain our findings should consider all the above factors to decipher the mechanism underlying the high levels of SCFAs in diarrheal samples. In vitro models mimicking colon conditions could help demonstrate the effect of SCFAs produced by the gut microbiota on DEC pathogenesis and clarify the impact of high SCFAs levels on diarrhea caused by DEC.

This study aimed to investigate the metabolic environment associated with DEC pathogenicity and measure the levels of key SCFAs in the gut. The results showed that higher levels of acetate, propionate, and butyrate were associated with DEC-positive diarrhea compared to healthy stools. Additionally, correlations were found between genera and their SCFAs production, and a higher redundancy of genomes was observed in diarrheal samples than in the healthy group. This study might help decipher the molecular mechanisms underlying DEC infection and enable strategies for preventing or treating diarrhea.

## MATERIALS AND METHODS

### Patients and Samples

Stool samples obtained from children under 5 years old with acute diarrhea, defined as more than three liquid stools per day (WHO definition, [49]), admitted to Dr. Luis Calvo Mackenna Hospital (HLCM), and control stool samples obtained from healthy children attending the HLCM daycare center were collected during 2019–2020. The stool samples were tested for enteropathogen identification using a FilmArray® gastrointestinal panel (FilmArray® GI) [50]. FilmArray® GI is an FDA-cleared qualitative syndromic PCR system that detects the following gastrointestinal pathogens: viruses (Adenovirus F40/41; Astrovirus; Norovirus GI/GII; RotavirusA; and Sapovirus I, II, IV and V), bacteria (*C. jejuni*; *C. coli*; *C. upsaliensi*s; *C. difficile*; *P. shigelloides*; *Salmonella*; *Yersinia enterocolitica*; *V. parahaemolyticus*; *V. vulnificus*; *V. cholera*; *Shigella*; and DEC pathotypes EAEC, EPEC, ETEC, STEC, and EIEC), and parasites (*Cryptosporidium*; *C. cayetanensis*; *E. histolytica*; and *G. lamblia*). After FilmArray® GI testing, all the stool samples were kept at -80°C. In this study, 83 samples were used, including 40 diarrheal samples that were only positive for one DEC pathotype (DEC group) and age-matched control samples (Healthy group) that were negative for the presence of pathogens, as well as three additional samples from this healthy group **(Table 1)**. Every child in this study was a resident of Santiago, Chile, and they all ate a standard diet without being breastfed. Parents declared that their children had not consumed probiotics, prebiotics, or antibiotics in the two months prior to sample collection at the time of enrollment.

**Table 1.** Characteristics of the samples used in the study.

**Characteristics**	**DEC group**	**Healthy group**
Number of samples	40	43
Age in months (interquartile range)	36.5 (25–51)	36.7 (29 - 47)
Pathogen detected (number of samples)	STEC (11); EAEC (11); EPEC (10); ETEC (6); *Shigella/*EIEC (2)	None

### Ethics

This study was conducted in accordance with the Declaration of Helsinki guidelines. The Universidad de Chile Ethics Committee (No. 032-2020) approved the study protocol. Written informed consent was obtained from all the parents on behalf of their children.

### SCFAs quantification

A 500 mg aliquot of stool was diluted in molecular-grade distilled water and centrifuged at 5,000 x g for 15 min, and supernatants (fecal waters) were collected and filtered with a 0.2 μm filter. Fecal waters were used to determine acetate, propionate, and butyrate using high-pressure liquid chromatography (HPLC; Agilent Technologies Inc.) at the HLCM biochemistry laboratory, following a published protocol [51]. Briefly, 100 μL of concentrated HCl were added to 1 mL of fecal water, followed by a vortex mixing step of 15 s. The samples were extracted for 20 minutes (gently rolling) using 5 mL of diethyl ether. Next, samples were centrifuged for 5 min at 3500 rpm, and 500 μL of 1M solution of NaOH was added to the supernatant. Later, samples were extracted again for 20 min, followed by a centrifugation step. The aqueous phase was transferred to an autosampler vial, and 100 μL of concentrated HCl were added. After vigorous vortex mixing, a 20 μL aliquot was injected onto the HPLC-UV apparatus (Agilent Technologies series 1260 infinity), using a Hypersil Gold aQ column (150 mm×4.6 mm i.d.) with particle sizes of 3 μm (Sercolab, Merksem, Belgium). The mobile phase consisted of 20 mM of NaH_2_PO_4_ in HPLC water (pH 2.2) and acetonitrile. The UV detector was set at a wavelength of 210 nm. The dry weight of the pellets was used to normalize the levels of SCFAs, as reported [24], and results were expressed as μmol of SCFAs per gram of stool. The statistical analysis for each compound was conducted using the Mann-Whitney test to compare the DEC and healthy samples. A *p*-value <0.05 was considered statistically significant. All analyses were performed using GraphPad Prism software (GraphPad, GraphPad Inc., Pittsburgh, PA, USA).

### DNA Extraction and 16S rRNA gene sequencing

Total DNA was extracted from each sample (200 mg of stool) using the QIAamp Power Soil (Qiagen) [52], quantified using a Synergy HT® spectrophotometer (Biotek) [53], and stored at -20 °C. DNA samples were shipped to the Molecular Research DNA Laboratory (MrDNA Lab; TX, USA) for DNA amplification and sequencing of the V3-V4 regions of the 16S rRNA (primers 341F (CCTACGGGNGGCWGCAG) and 785R (GACTACHVGGGTATCTAATCC) [54]), using the Illumina MiSeq 2 × 300 PE. Raw Illumina data (without adapters) was processed locally as follows. Briefly, raw sequences were processed using QIIME2 [55] software and its default plugins: demux for demultiplexing sequences and q2-cutadapt for primer removal. The default denoising parameters of DADA2 [56] were used until the formation of amplicon sequence variants (ASVs) [57]. ASVs were taxonomically classified at the genus level using the SILVA REF138 database as a reference [58]. The presence of ASVs was scaled using a double square root to decrease the impact on the diversity of *E. coli* infection in the DEC group and normalized to relative abundance. The genera present in all samples were compared between groups using the Mann-Whitney U test, and untransformed data were evaluated using differential abundance with the analysis of the composition of microbiomes (ANCOM) [59] plugin of QIIME2 [55]. The *vegan*, *Philoseq*, *Microbiome,* and *ggplot2* packages [60–63] of R software [64] were used for correlation analysis, sample clustering, determination of alpha (Simpson, Shannon, richness indexes), and beta diversity (RDA) [65] analyses, and graph plotting. Correlations between the levels of detected SCFAs and the abundance of bacterial genera were determined using the *stats* package [64] from R. For correlations, samples without detected levels of SCFAs were excluded from the analysis; the genera not correlated with any of the detected SCFAs were not graphically represented.

### Shotgun Metagenomics

To identify specific bacterial species associated with the observed levels of SCFAs within each group, ASV abundance was used to select a subset of samples for shotgun metagenome sequencing. We selected 16 samples per group, using the following criteria: age between 36 and 60 months and detectable levels of SCFAs. First, samples were clustered based on microbiota composition using the *Manhattan* distance and *Ward2* [66] methods. Twenty samples that were the furthest from the opposite group were selected. Additionally, as supplementary criteria, only samples from children over 3 years were chosen because their microbiota has been described as more stable, and acetate levels are expected to be the most abundant in the gut. Furthermore, only samples with SCFA levels within physiological proportions were included. The final subset for metagenome analysis comprised 16 DEC samples and 16 control samples, and the final subset for metagenome analysis included 16 DEC samples and 16 control samples. To evaluate the representativeness of the original samples and subgroups, samples were clustered again based on microbiota composition, and SCFA levels between DEC and healthy subgroups were compared. Shotgun metagenomic sequencing of the subgroup of 32 samples was performed using a 2 × 150 bp PE MiSEQ Illumina (MrDNA Lab). Raw data was trimmed to maintain the minimum length of sequences at 100 bp using FastQC [67] and *bbduk*(ktrim=r, k=28, mink=12, hdist=1, tbo=t, tpe=t, qtrim=rl, trimq=20, minlength=100) [68]. Trimmed data was coupled using the Enveomics metagenomic package (∼/enveomics/Scripts/FastA.interpose.pl) [69] and finally assembled using IDBA_ud (∼/idba-master/bin/idba_ud) [70] with a minimum contig size of 500 bp. The quality of the assemblies was determined using the N50 parameter and Nonpareil [71]. MaxBin2 [72] was used for binning sequences, with a minimum contig length of 2,000 bp. Raw 16S rRNA sequencing and metagenomics data have been published in the Ebi-ENA repository under the ERP146121 project number.

### Species determination and mapping

Metagenomes were completely assembled to determine the species present in the samples and to evaluate the presence of genes associated with the production of acetate, propionate, and butyrate. The quality of the metagenome-assembled genomes (MAGs) was evaluated using CheckM [73], and only MAGs with completeness above 60% and contamination below 10% were maintained. As a final step in the selection, MiGA@XSEDE [74] was used to dereplicate the MAGs. Finally, GTDBTk [75] was used for the taxonomic affiliation of MAGs. Metagenomic reads were mapped to dedicated MAGs using BLASTn. Reads mapping at >95% similarity and 70% coverage were used for the MAG abundance analysis, and the value was normalized by the average genome size, determined using MicrobeCensus [76], and then normalized by percentage. Metorigin [77] was used to evaluate the associations between MAGs and metabolic pathways involved in the detected levels of SCFAs. Additionally, we used Prodigal [78] to predict coding sequences from non-repetitive MAGs and utilized BLAST+ [79] to align them with target genes. Gene sequences related to the generation of acetate, propionate, and butyrate were acquired from the UniProt repository [80]. The *ackA* gene was assessed for its involvement in acetate production, whereas the *mmdA*, *lcdA*, and *pduP* genes were identified for their role in propionate production. The *but* and *buk* genes were mapped to determine their role in butyrate synthesis, while the *rpoB* gene was employed as a reference gene [81].

## SUPPLEMENTAL MATERIAL

Click here for supplemental data file.

All supplemental data for this article are available online at www.microbialcell.com/researcharticles/2024a-gallardo-microbial-cell/.

## Abbreviations:

DEC: – diarrheagenic *Escherichia coli*,
SCFAs: – Short-chain fatty acids,
MAGs: – metagenome-assembled genomes,
ASVs: – amplicon sequence variants,
IBS: – irritable bowel syndrome,
HPLC: – using high-performance liquid chromatography,
RDA: – redundancy analysis,
GPR: – G protein-coupled receptors,
TLR: – toll-like receptors,
HLCM: – Dr. Luis Calvo Mackenna Hospital.
