# Supplementary material for: A metagenomic approach to unveil the association between fecal gut microbiota and short-chain fatty acids in diarrhea caused by diarrheagenic Escherichia coli in children
Source: Microb Cell. 2024 Apr 30;11:116–27. doi: 10.15698/mic2024.04.820 (PMC11122282; doi:10.15698/mic2024.04.820)
Supplement: Supplementary file 1 [file mic-11-116-s01.pdf]

Supplementary Figures

Supplementary Figure 1. Levels of SCFAs within DEC samples. Concentrations of acetate, propionate, and butyrate in stool samples based on pathotypes. Age-paired DEC-positive samples are represented by red dots and grouped by the pathotype, while green dots represent healthy samples. Values are normalized by dry pellet weight. Analysis was performed using the Mann-Whitney test. The *p*-values for each SCFA are displayed in the figure. The number of samples (n) included in each group is stated in parentheses.

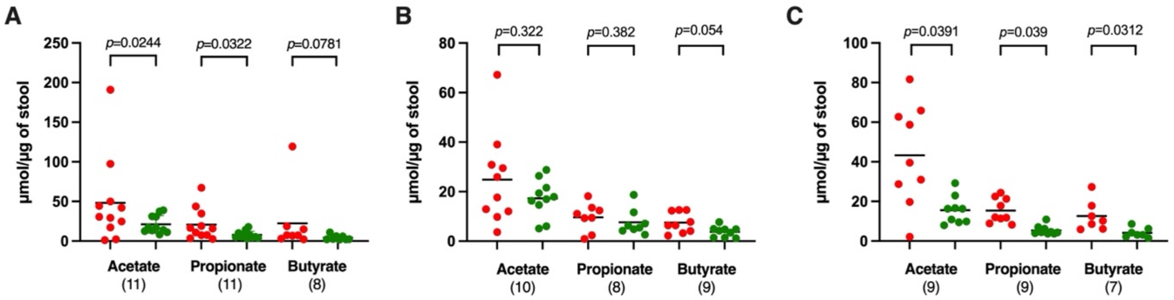

Supplementary Figure 2. Ratio of *Firmicutes*/*Bacteroidota*. The graph displays the *Firmicutes/Bacteroidota* relative abundance ratio in each stool sample, as determined using 16S rRNA sequencing. Red dots represent the DEC samples, while green dots represent the healthy samples. Analysis was performed using the Mann-Whitney test.

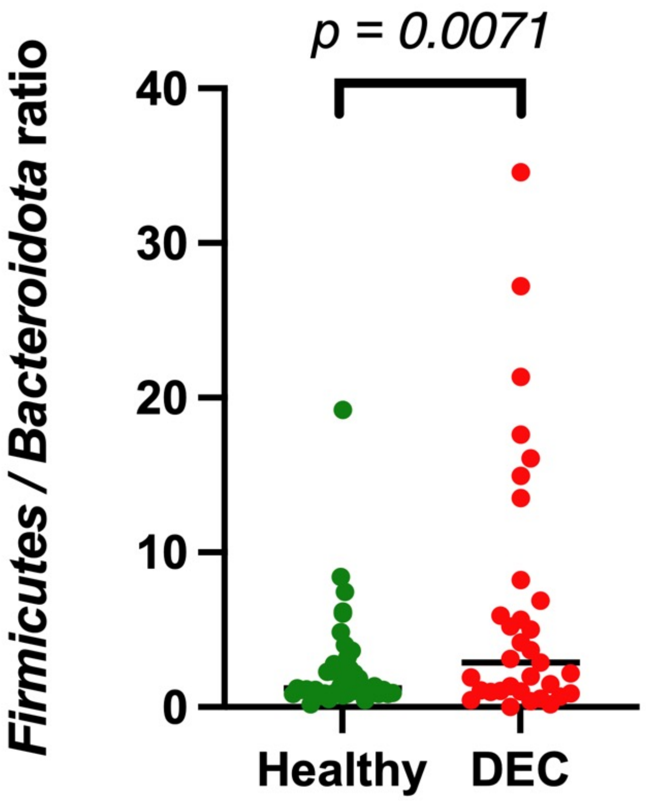

Supplementary Figure 3. A) Relative abundance of significant genera. The significant genera shown in Figure 2B have been plotted here to show the dispersion of samples among groups. Significance from the original comparison is also indicated here (**$p < 0.01$, *$p < 0.05$). **B) Analysis of Compositions of Microbiomes (ANCOM).** Differential analysis of microorganisms using the ANCOM method. The x-axis value represents the centered log-ratio transformed mean difference in abundance of genera between the healthy and DEC-positive group, with healthy-associated bacteria on the left (negative) side and DEC-associated organisms on the right (positive) side. The W value on the y-axis represents the number of times the null hypothesis was rejected for a given species. Genera with a reject null hypothesis>95% are colored in bold according to the group they are associated with; other genera, found significantly associated with either the DEC or healthy group, using non-parametric methods, are soft colored.

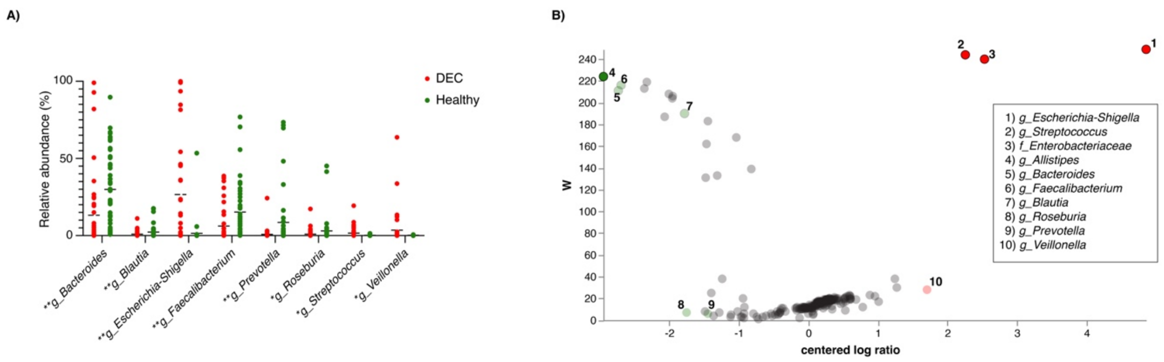

Supplementary Figure 4. Dendrogram based on microbiota composition of subgroups of samples selected for metagenomes. Distances were calculated using double-square transformed ASV abundance from 16S rRNA amplicons, using Manhattan distance and Ward.D2 clustering.

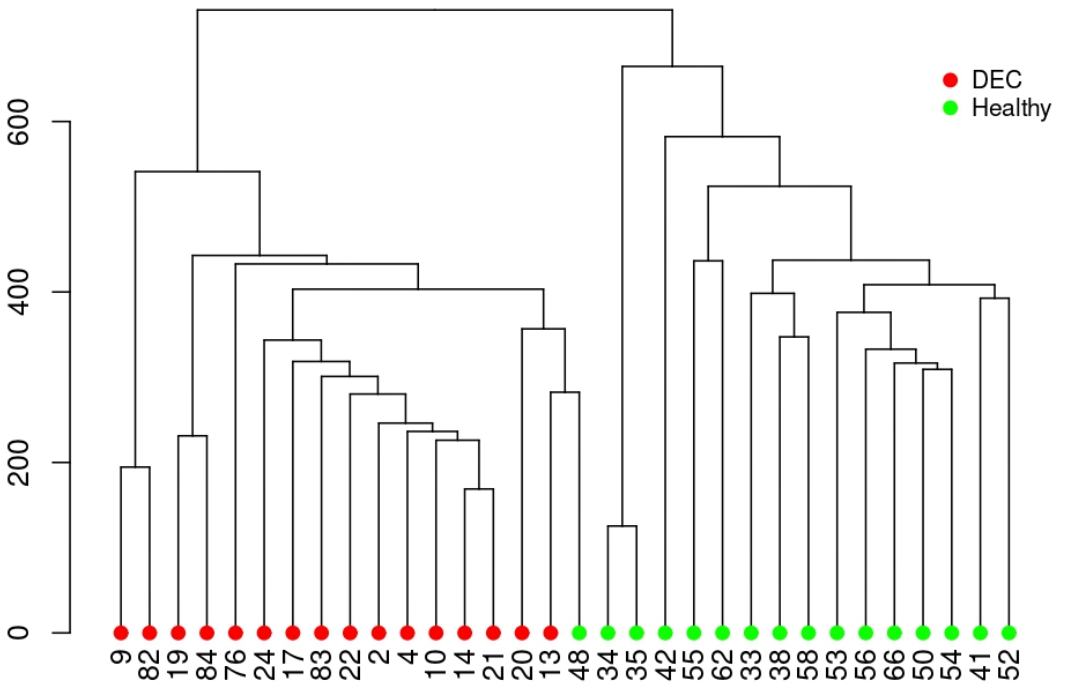

Supplementary Figure 5. Levels of SCFAs in subgroups of stool samples. HPLC-detected acetate, propionate, and butyrate levels in the stool samples selected for metagenome analysis. Red dots represent DEC-positive samples, and green dots are healthy samples. Values are normalized by dry pellet weight. Analysis was performed using the Mann-Whitney test. The *p*-values for each SCFA are displayed in the figure.

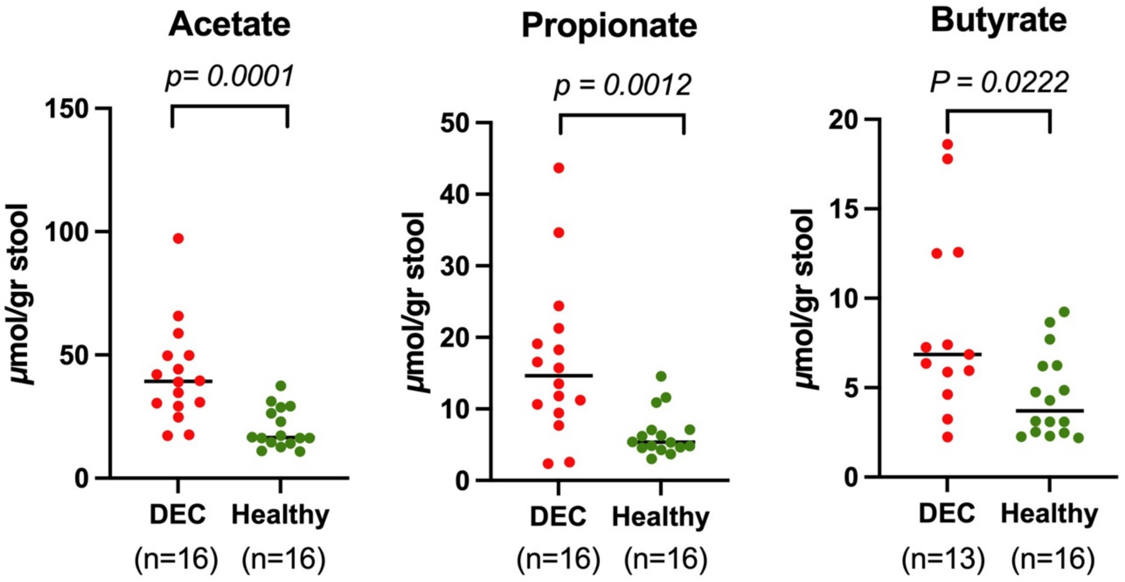

Supplementary Figure 6. Level of Coverage in Metagenomic Datasets. Diversity (**A**) from metagenome sequencing, *kappa* index, coverage with the respective fitting model **(B),** and sequencing effort **(C)** was calculated using Nonpareil. Red dots represent data values for DEC samples and green for healthy samples. Yellow dots represent the predicted sequencing effort to reach 95 % coverage, and blue dots represent the actual values.

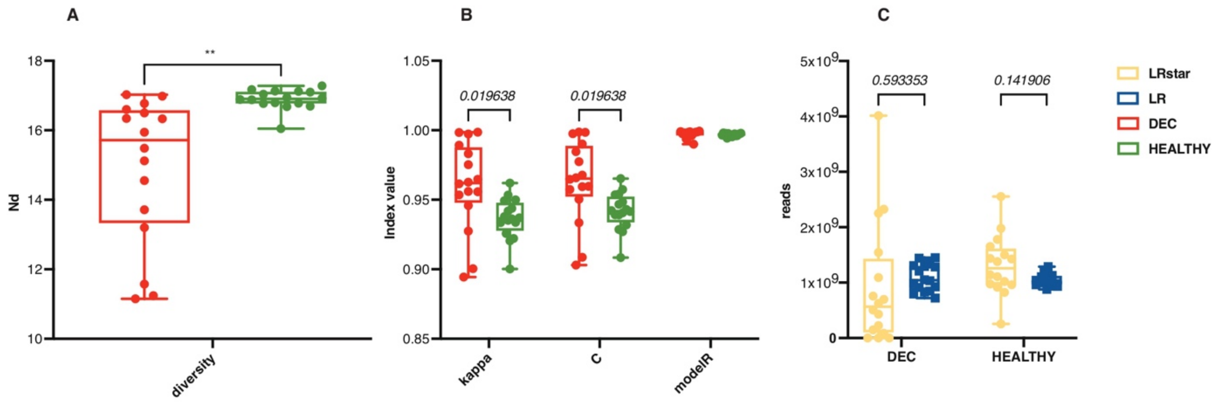

Supplementary Figure 7. Summary of network co-metabolism in DEC samples at genera level. The network illustrates the relationships between bacteria and significant SCFAs in DEC samples. The circles in the diagram symbolize bacteria. The red circles indicate highly abundant bacteria, with a soft red color indicating non-significant abundance and bold red indicating significant abundance. The green circles represent a decrease in bacterial presence, with a soft green color indicating a non-significant decrease and bold green indicating a significant reduction when comparing DEC samples to healthy individuals. Diamonds symbolize metabolites (bold red for significant presence). The biological associations are represented by red and green lines, with red indicating positive and green indicating negative associations. Soft lines suggest non-significant associations, while strong lines imply significant associations.

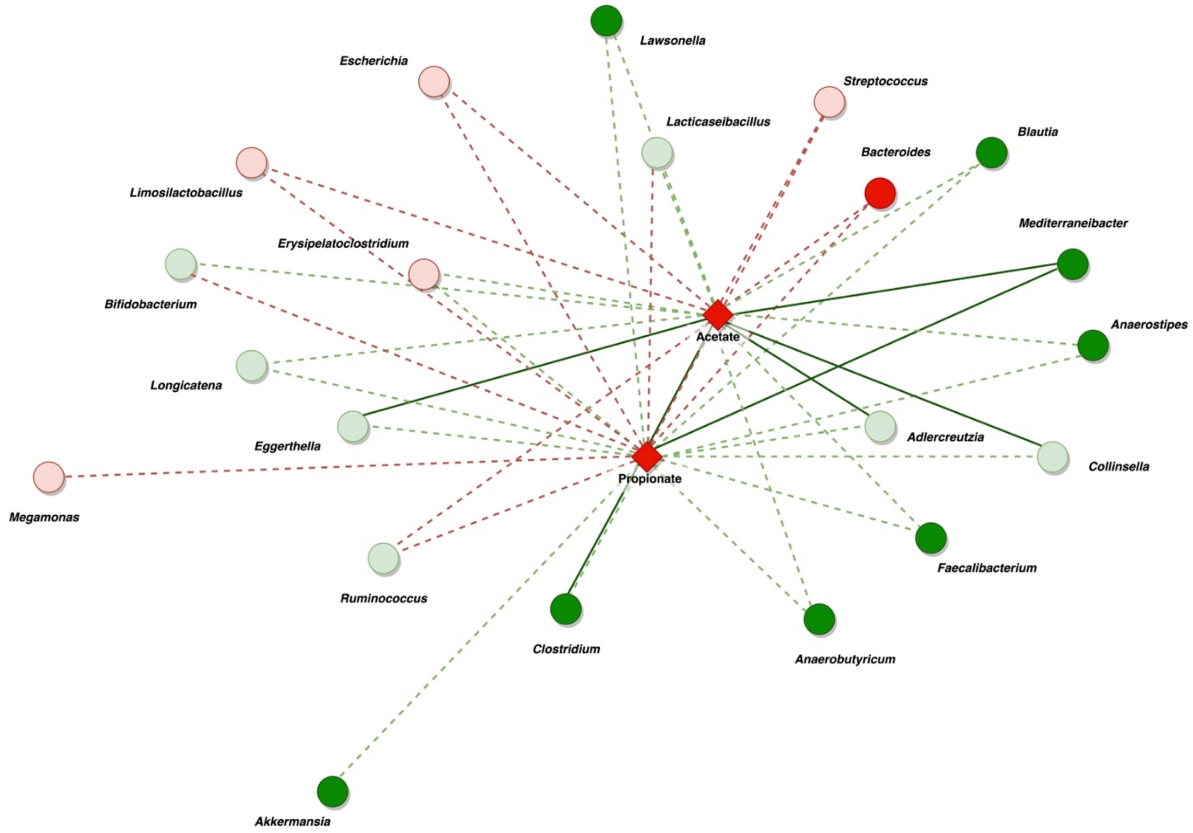

Supplementary Figure 8. Ratio of the abundance of genes involved in the final steps of production of acetate (*ackA*), propionate (*mmdA, lcdA, pduP*), and butyrate (*but*, *buk*) in MAGs. (A) The ratio of individual genes vs. presence of housekeeping *rpoB*. **(B)** The ratio of grouped genes vs. presence of housekeeping *rpoB*. **(C)** Ratio of grouped genes vs. reads per million (RPM).

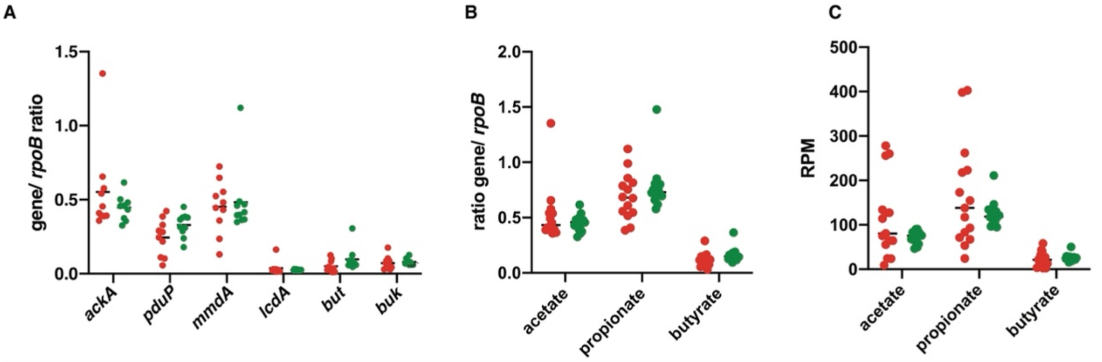

Supplementary Tables

Supplementary Table 1: Quality report of raw processing of 16S data.

sample-id	input	filtered	percentage of input passed filter (%)	denoised	merged	percentage of imput merged (%)	non-quimeric	percentage of imput non-chimeric (%)
1	52480	45695	87.07	45470	44303	84.42	27706	52.79
2	42731	37614	88.03	37124	36045	84.35	22320	52.23
3	56256	49857	88.63	49395	47718	84.82	24047	42.75
4	61452	47250	76.89	46543	44756	72.83	34210	55.67
5	33035	29190	88.36	27674	24054	72.81	14708	44.52
6	40460	35901	88.73	35112	31612	78.13	19021	47.01
7	57975	51184	88.29	51039	50888	87.78	49303	85.04
8	34474	30597	88.75	27782	22587	65.52	10654	30.9
9	36885	28003	75.92	27000	24968	67.69	13023	35.31
10	22054	19480	88.33	18731	17563	79.64	13304	60.32
11	40901	20665	50.52	19848	17279	42.25	8942	21.86
12	64232	53705	83.61	53383	53079	82.64	45571	70.95
13	35937	32158	89.48	31285	28807	80.16	17138	47.69
14	42777	29945	70	29129	28133	65.77	24140	56.43
15	33098	29073	87.84	29022	28619	86.47	21492	64.93
16	35004	28149	80.42	27815	27566	78.75	25940	74.11
17	58931	51863	88.01	51020	47979	81.42	26172	44.41
18	28108	22988	81.78	22491	21566	76.73	15877	56.49
19	37376	33485	89.59	32983	32035	85.71	26510	70.93
20	36034	30178	83.75	28837	26462	73.44	15866	44.03
21	39747	34910	87.83	34778	34592	87.03	33684	84.75
22	30438	22455	73.77	21590	19872	65.29	12603	41.41
23	25025	22542	90.08	20293	15167	60.61	8166	32.63
24	37709	33098	87.77	31852	29239	77.54	19971	52.96
25	33751	28718	85.09	27943	26154	77.49	17972	53.25
26	38703	33698	87.07	33147	31558	81.54	19515	50.42
27	39181	31483	80.35	31071	30362	77.49	29525	75.36
28	26117	23397	89.59	23016	21738	83.23	12420	47.56
29	30205	25152	83.27	24379	22320	73.9	10855	35.94
30	41566	37311	89.76	36480	33660	80.98	19252	46.32
31	21911	16999	77.58	15848	12812	58.47	4530	20.67
32	29209	26353	90.22	24838	21023	71.97	6228	21.32
33	30022	24527	81.7	23505	19935	66.4	8442	28.12
34	39459	32904	83.39	30702	25934	65.72	13865	35.14
35	23821	21467	90.12	19778	16616	69.75	9936	41.71
36	25874	23418	90.51	22588	19966	77.17	7739	29.91

37	37802	30844	81.59	29241	25408	67.21	11815	31.25
38	23634	20833	88.15	19238	15284	64.67	6781	28.69
39	29449	23834	80.93	21049	14965	50.82	6064	20.59
40	29799	24754	83.07	23417	19708	66.14	8815	29.58
41	45580	36526	80.14	35229	31658	69.46	17100	37.52
42	39578	30676	77.51	28651	25085	63.38	18535	46.83
43	39826	33021	82.91	30596	24548	61.64	11677	29.32
44	23477	19850	84.55	18604	16073	68.46	8541	36.38
45	37960	31156	82.08	29299	24840	65.44	13730	36.17
46	21338	19071	89.38	18160	15360	71.98	5295	24.81
47	51534	42345	82.17	39473	31539	61.2	12899	25.03
48	27506	25039	91.03	23921	22308	81.1	14131	51.37
49	44371	33898	76.4	32521	29609	66.73	17065	38.46
50	54175	46635	86.08	45103	42996	79.37	37505	69.23
51	25194	22088	87.67	20185	16784	66.62	9867	39.16
52	36176	31507	87.09	30295	27306	75.48	16677	46.1
53	39329	34731	88.31	32613	28306	71.97	14569	37.04
54	24879	21405	86.04	19992	18032	72.48	13098	52.65
55	51660	44993	87.09	43076	38929	75.36	24698	47.81
56	30397	23510	77.34	22539	21154	69.59	18701	61.52
57	31754	27815	87.6	26614	23534	74.11	13507	42.54
58	42321	33726	79.69	32191	29389	69.44	18478	43.66
59	39324	35451	90.15	34732	32300	82.14	18904	48.07
60	46127	41706	90.42	40287	36293	78.68	11161	24.2
61	21934	19273	87.87	17282	14096	64.27	8739	39.84
62	26873	23747	88.37	22490	19171	71.34	12220	45.47
63	29095	24660	84.76	23481	20729	71.25	11331	38.94
64	26868	21194	78.88	20077	17475	65.04	11361	42.28
65	25360	23057	90.92	22550	21288	83.94	15302	60.34
66	24665	21561	87.42	20107	16373	66.38	8529	34.58
67	27457	24192	88.11	21479	16848	61.36	9433	34.36
69	53043	41844	78.89	41140	38312	72.23	23410	44.13
70	37625	29243	77.72	27885	24270	64.5	16044	42.64
72	41199	34677	84.17	34403	32831	79.69	18134	44.02
73	29602	26063	88.04	25912	25662	86.69	24678	83.37
74	29657	26605	89.71	26534	26025	87.75	10650	35.91
75	59552	50038	84.02	49783	48587	81.59	10667	17.91
76	45432	35262	77.61	33681	28044	61.73	12445	27.39
77	47910	34527	72.07	33511	28695	59.89	14027	29.28
78	30064	14409	47.93	13942	13151	43.74	9909	32.96
79	45867	34254	74.68	32856	28079	61.22	13406	29.23
80	20920	18776	89.75	18312	17507	83.69	12700	60.71

81	21478	18790	87.48	17697	15010	69.89	9001	41.91
82	46079	36318	78.82	35375	32781	71.14	16921	36.72
83	29225	26146	89.46	25437	23539	80.54	15869	54.3
84	32770	29556	90.19	28718	26917	82.14	21002	64.09
85	39348	30074	76.43	29710	28600	72.68	19661	49.97

Supplementary Table 2: List of recovered MAGs from each group.

DEC group			Healthy group	
Ref	GDTB v207 classification		Ref	GDTB v207 classification
GCA_900543555.1	*s__Acutalibacter sp900543555*		N/A	*g__Lawsonella*
GCF_000025985.1	*s__Bacteroides fragilis*		N/A	*g__Collinsella*
GCF_001025175.1	*s__Bifidobacterium breve*		GCF_900048895.1	*s__Acutalibacter timonensis*
GCF_000196555.1	*s__Bifidobacterium longum*		GCF_003340305.1	*s__Adlercreutzia hattorii*
GCF_001025215.1	*s__Bifidobacterium pseudocatenulatum*		GCF_000020225.1	*s__Akkermansia muciniphila*
GCF_001487165.1	*s__Blautia_A massiliensis*		GCF_900209925.1	*s__Anaerobutyricum hallii_A*
GCF_000153905.1	*s__Blautia_A obeum*		GCF_000332875.2	*s__Anaerostipes hadrus*
GCA_900066205.1	*s__Blautia_A sp900066205*		GCF_000010425.1	*s__Bifidobacterium adolescentis*
GCF_000484655.1	*s__Blautia_A wexlerae*		GCF_001025135.1	*s__Bifidobacterium bifidum*
GCF_003460745.1	*s__CAG-41 sp900066215*		GCF_001042615.1	*s__Bifidobacterium kashiwanohense*
GCA_900540255.1	*s__Clostridium sp900540255*		GCF_000196555.1	*s__Bifidobacterium longum*
GCF_005844325.1	*s__Collinsella aerofaciens_G*		GCF_001025215.1	*s__Bifidobacterium pseudocatenulatum*
GCF_000225705.1	*s__Collinsella tanakaei*		GCA_005844445.1	*s__Blautia_A caecimuris*
GCF_000024265.1	*s__Eggerthella lenta*		GCF_001487165.1	*s__Blautia_A massiliensis*
GCF_000154485.1	*s__Erysipelatoclostridium ramosum*		GCA_900066205.1	*s__Blautia_A sp900066205*
GCF_000026325.1	*s__Escherichia coli_D*		GCF_000484655.1	*s__Blautia_A wexlerae*
GCF_001405555.1	*s__Fusicatenibacter saccharivorans*		GCA_000436335.1	*s__CAG-217 sp000436335*
GCF_000829035.1	*s__Lacticaseibacillus paracasei*		GCF_003460745.1	*s__CAG-41 sp900066215*
GCF_001436025.1	*s__Limosilactobacillus mucosae*		GCA_000435335.1	*s__CAG-964 sp000435335*
GCF_004341945.1	*s__Longicatena caecimuris*		GCA_900541285.1	*s__Collinsella sp900541285*
GCF_000371425.1	*s__Longicatena innocuum*		GCA_900541745.1	*s__Collinsella sp900541745*
GCF_900186585.1	*s__Massilimaliae timonensis*		N/A	*s__Copromorpha sp900545775*
GCF_000245775.1	*s__Megamonas funiformis*		GCF_000024265.1	*s__Eggerthella lenta*
GCF_000147675.1	*s__Peptostreptococcus stomatis*		GCF_003425665.1	*s__Evtepia gabavorous*
GCF_900537995.1	*s__Roseburia intestinalis*		GCF_002550035.1	*s__Faecalibacterium prausnitzii_E*

GCF_002834235.1	*s__Ruminococcus_E bromii_B*		GCF_001405555.1	*s__Fusicatenibacter saccharivorans*
GCF_001280875.1	*s__Sellimonas intestinalis*		GCF_900167555.1	*s__Gemmiger formicilis*
GCF_000785515.1	*s__Streptococcus salivarius*		GCF_003324125.1	*s__Gemmiger qucibialis*
GCF_000187445.1	*s__Streptococcus sp000187445*		GCA_900539695.1	*s__Gemmiger sp900539695*
			GCA_001312505.1	*s__Mediterraneibacter faecis*
			GCF_004123145.1	*s__Oliverpabstia faecicola*
			GCF_002834235.1	*s__Ruminococcus_E bromii_B*
			GCA_900545085.1	*s__Scybalosoma faecavium*
			GCA_003531055.1	*s__UBA1417 sp003531055*
